# Effects of cannabidiol on anandamide levels in individuals with cannabis use disorder: findings from a randomised clinical trial for the treatment of cannabis use disorder

**DOI:** 10.1038/s41398-023-02410-9

**Published:** 2023-04-21

**Authors:** Daniel Ying-Heng Hua, Chandni Hindocha, Gianluca Baio, Rachel Lees, Natacha Shaban, Celia J. Morgan, Ali Mofeez, H. Valerie Curran, Tom P. Freeman

**Affiliations:** 1 Addiction and Mental Health Group (AIM), Department of Psychology, University of Bath, Bath, UK; 2 Clinical Psychopharmacology Unit, UCL, London, UK; 3 University College London, London, UK; 4 University of Oxford, Oxford, UK; 5 Washington Singer Labs, University of Exeter, Exeter, UK; 6 Pain Management Centre, National Hospital for Neurology and Neurosurgery, UCLH, London, UK

**Keywords:** Clinical pharmacology, Human behaviour

## Abstract

Cannabidiol (CBD) has shown promise in treating psychiatric disorders, including cannabis use disorder – a major public health burden with no approved pharmacotherapies. However, the mechanisms through which CBD acts are poorly understood. One potential mechanism of CBD is increasing levels of anandamide, which has been implicated in psychiatric disorders including depression and cannabis use disorder. However, there is a lack of placebo-controlled human trials investigating this in psychiatric disorders. We therefore assessed whether CBD affects plasma anandamide levels compared to placebo, within a randomised clinical trial of CBD for the treatment of cannabis use disorder. Individuals meeting criteria for cannabis use disorder and attempting cannabis cessation were randomised to 28-day administration with placebo (*n* = 23), 400 mg CBD/day (*n* = 24) or 800 mg CBD/day (*n* = 23). We estimated the effects of each CBD dose compared to placebo on anandamide levels from baseline to day 28. Analyses were conducted both unadjusted and adjusted for cannabis use during the trial to account for effects of cannabis on the endocannabinoid system. We also investigated whether changes in plasma anandamide levels were associated with clinical outcomes relevant for cannabis use disorder (cannabis use, withdrawal, anxiety, depression). There was an effect of 800 mg CBD compared to placebo on anandamide levels from baseline to day 28 after adjusting for cannabis use. Pairwise comparisons indicated that anandamide levels unexpectedly reduced from baseline to day 28 in the placebo group (−0.048, 95% CI [−0.089, −0.007]), but did not change in the 800 mg CBD group (0.005, 95% CI [−0.036, 0.047]). There was no evidence for an effect of 400 mg CBD compared to placebo. Changes in anandamide levels were not associated with clinical outcomes. In conclusion, this study found preliminary evidence that 28-day treatment with CBD modulates anandamide levels in individuals with cannabis use disorder at doses of 800 mg/day but not 400 mg/day compared to placebo.

## Introduction

In recent decades, there has been considerable interest in the use of cannabinoids for medicinal purposes [1]. The most abundant cannabinoids are ∆^9^-tetrahydrocannabinol (THC) and cannabidiol (CBD) [2, 3]. Unlike THC, CBD is non-intoxicating and has few adverse effects at typical dosages [4]. CBD is recommended as a treatment for Lennox-Gastaut syndrome and Dravet syndromes [5]. A growing literature suggests that CBD may be a promising pharmacological intervention for several other indications.

### Efficacy of CBD in psychiatric disorders

Clinical studies have provided evidence on the efficacy of CBD for psychiatric disorders including anxiety, psychosis and cannabis use disorder. In a randomised controlled trial, an acute oral 600 mg dose of CBD performed better than placebo at reducing anxiety in individuals with social phobia during a public speaking task [6]. For psychosis, three randomised clinical trials of CBD have been conducted. Two of these demonstrated reductions in psychotic symptoms following CBD treatment when compared to placebo or active control, while the third showed no difference relative to placebo [7–9]. Lastly, regarding cannabis use disorder, this psychiatric disorder represents a growing public health issue. In the past two decades, the proportion of people seeking treatment for cannabis use disorders has risen in all world regions other than Africa. In Europe, the number of first-time clients presenting at addiction services for cannabis-related issues has increased by 76% in the past decade [10]. Importantly, unlike depression and psychosis, there are no pharmacotherapies approved for the treatment of cannabis use disorder. CBD has the potential to be a safe and efficacious treatment for this large unmet clinical need, for example by counteracting somatic and psychological symptoms of cannabis withdrawal, and decreasing attentional bias towards cannabis-related stimuli [11–13]. A clinical trial found that CBD at daily doses of 400 mg or 800 mg was more efficacious than placebo at reducing cannabis use among individuals meeting criteria for cannabis use disorder [14]. Such daily doses of CBD are considerably higher than those present in street cannabis, which predominantly contain THC and little if any CBD [15]. While further evidence is needed, existing data suggest that CBD could have a potential role in treating a range of psychiatric disorders.

### Therapeutic mechanisms of CBD

Despite interest in the potential medicinal uses of CBD [1], its therapeutic mechanisms of action are not well understood. Gaining further insight in this area may be important to further understanding the aetiology and treatment of psychiatric disorders including targets for translational models. CBD has a broad range of pharmacological mechanisms. For example, CBD has been demonstrated to affect proxy measures of glutamate and GABA in typically developing and autistic adults [16]. CBD may also act within dopaminergic, serotoninergic and endocannabinoid systems [17]. Of particular relevance may be the endocannabinoid system, as it has been implicated in a number of psychiatric disorders that CBD has shown therapeutic potential.

The endocannabinoid system consists of cannabinoid type 1 receptors (CB1Rs), type 2 receptors (CB2Rs), and endocannabinoid ligands that bind to these receptors such as anandamide and 2-arachidonoylglycerol (2-AG) [18]. Anandamide is a partial agonist of CB1Rs and is degraded by fatty acid amide hydrolase (FAAH), while 2-AG is a full agonist of CB2Rs that is primarily degraded by monoacylglycerol lipase (MAGL) [19].

Like CBD, the endocannabinoid system has been implicated in a range of psychiatric disorders. In mouse and rodent models, the experimental elevation of anandamide via exercise or inhibition of FAAH resulted in anxiolytic effects, which were then nullified by CB1R blockade [20, 21]. In humans, carriers of the FAAH 385A allele, which results in destabilised FAAH proteins and elevated anandamide levels, have been found to self-report lower anxiety compared to non-carriers [22]. Anandamide levels in plasma have also been found to be reduced in individuals with depression compared to healthy, matched controls [23].

In addition to anxiety and depression, the endocannabinoid system plays a key role in addiction [24]. Cannabis use disorder can occur from persistent heavy cannabis use, which causes an adaptive downregulation of brain endocannabinoid signalling [25]. Indeed, individuals who use cannabis more than ten times per month have been found to have reduced anandamide levels in cerebrospinal fluid compared to non-users [26]. Similarly, CB1R availability has been found to be reduced in daily cannabis users compared to healthy controls [27, 28]. Moreover, lower CB1R availability has been found to predict greater cannabis withdrawal severity [29], although not in all studies [27]. Increasing endocannabinoid signalling may therefore be a relevant therapeutic target when addressing cannabis use disorder. A recent phase 2a clinical trial of a FAAH inhibitor supports this, as compared to placebo, it was found to increase anandamide levels and decrease cannabis use and withdrawal symptoms among individuals with cannabis use disorder [30].

### Effects of CBD on the endocannabinoid system in psychiatric disorders

The body of evidence discussed thus far implicates the endocannabinoid system in a range of psychiatric disorders where CBD may have therapeutic potential. It is therefore plausible that the therapeutic effects of CBD occur, at least in part, through its actions on the endocannabinoid system. CBD may act on the endocannabinoid system through several means: by inhibiting lipoxygenases involved in anandamide degradation; [31] by competitively binding to proteins that transport anandamide towards FAAH enzymes for catabolism; [32] by mediating agonism bias of CB1Rs and CB2Rs that changes the effect of anandamide [33]. Repeated administration of CBD after a daily stressor has been found to approximately double anandamide levels within mouse hippocampi [34]. In humans, one randomised, double-blind trial of CBD among individuals with acute schizophrenia found that a dosage of 800 mg/day CBD for 28 days produced significantly increased anandamide levels when compared to a parallel group provided 800 mg/day amisulpride, or when compared to baseline [8]. Furthermore, increases in plasma anandamide levels following a course of CBD were correlated with a reduction in psychotic symptoms [8].

Although this preliminary evidence indicates an effect of CBD within the endocannabinoid system, our mechanistic understanding of this pathway is impeded due to methodological limitations of the current literature. While animal studies offer insight into the preclinical pharmacology of CBD, it remains to be seen whether CBD causes the same effects in humans. While Leweke et al. [8] provide the first indication that CBD increases anandamide levels in humans, this trial was not placebo-controlled and instead compared CBD to amisulpride, a first-line antipsychotic. It is therefore unclear to what extent CBD compared to placebo affects anandamide levels in humans.

### The current study

To our knowledge, the current study is the first to investigate the effects of CBD on anandamide levels compared to placebo in the treatment of a psychiatric disorder. These data were recorded from plasma measures of anandamide as part of a randomised clinical trial of CBD for cannabis use disorder [14]. The primary analysis from the trial demonstrated that 400 mg/day and 800 mg/day oral CBD for 28 days was more efficacious than placebo at reducing cannabis use. Here we present data on the effects of 400 mg/day and 800 mg/day CBD on plasma measures of anandamide and their association with anxiety, depression, cannabis use and withdrawal symptoms during a 28-day course of CBD.

## Method

### Study design

**Fig. 1:**
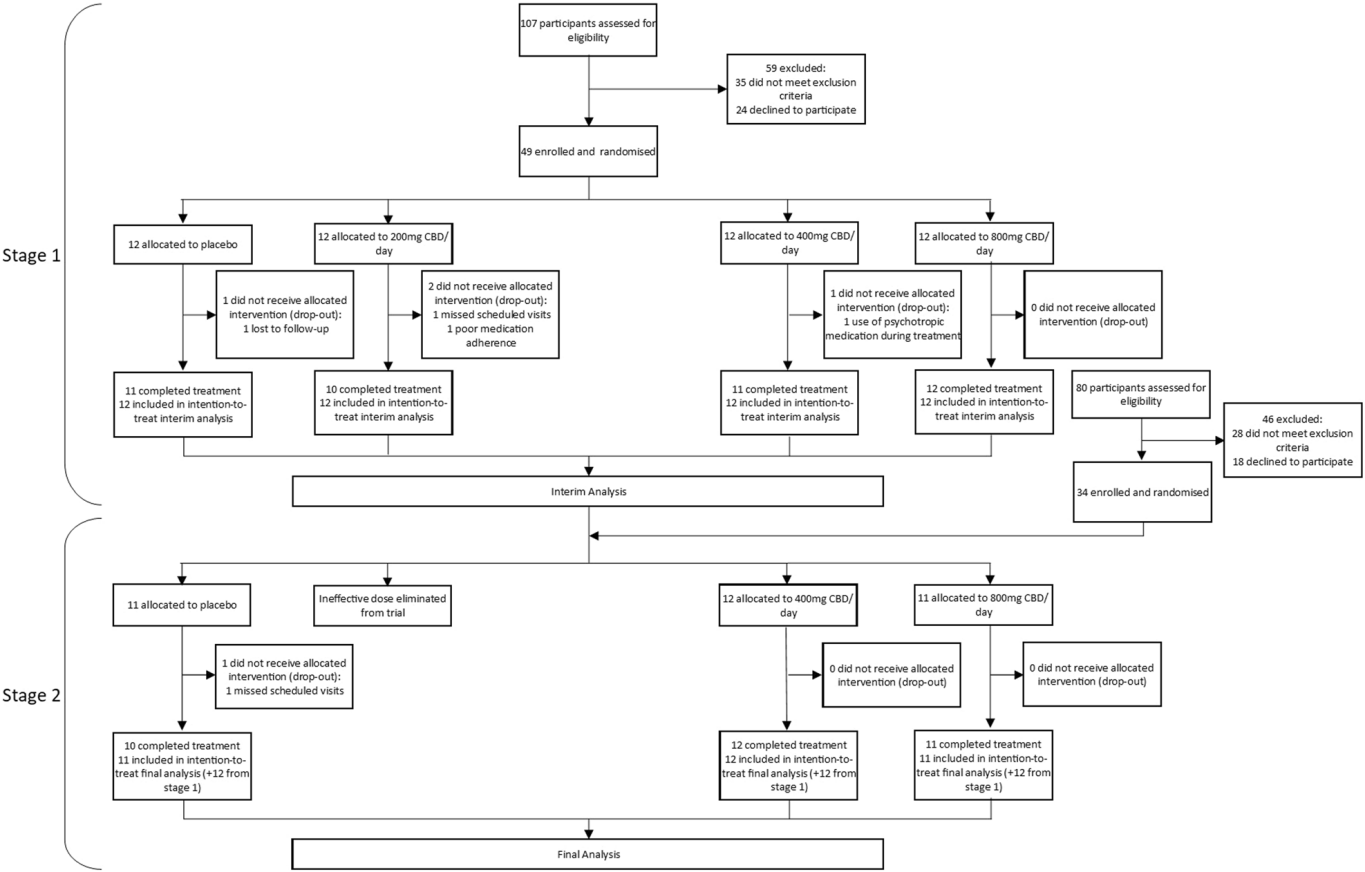
CONSORT diagram. Diagram showing enrolment, allocation, and the groups at each stage of analysis.

A randomised, double-blind, placebo-controlled phase 2a clinical trial was conducted to investigate CBD as a treatment for cannabis use disorder. The full methodology of this trial, including randomisation and blinding, has been reported previously [14]. This was a phase 2a trial using a two-stage adaptive Bayesian dose-finding design, meaning that multiple dosages were used in order to statistically analyse the most and least efficacious dosage at mid-trial (stage 1) and end of trial (stage 2). Least efficacious dosages were eliminated at stage 1 so that subsequently recruited participants could be allocated to more promising dosages, which utilised resources and participants more efficiently. Following screening, participants attended a baseline visit (day 0). At the end of their baseline visit, participants began a cannabis cessation attempt and were randomised to parallel treatment arms lasting 28 days. This length of time was chosen based on a previous trial showing that 800 mg CBD was well tolerated and associated with improved clinical symptoms after 28 days of treatment, with good tolerability [8]. During stage 1 of the adaptive trial, participants were randomised to parallel treatment arms of CBD (200 mg/day, 400 mg/day, 800 mg/day) or placebo, using a 1:1:1:1 allocation ratio (see Fig. 1 for number of participants and drop-out rates in each treatment arm). These dosages were chosen as previous studies have shown repeated doses of 200–800 mg CBD to have excellent tolerability and safety [35]. Following a planned interim analysis, 400 mg CBD/day and 800 mg CBD/day were deemed more efficacious than placebo at reducing cannabis use. By contrast, the 200 mg CBD/day dose was deemed inefficacious via planned Bayesian analyses [14] and this dosage was eliminated from the trial, with newly recruited participants being randomly and equally allocated to the remaining three treatment arms at stage 2. The 200 mg/day CBD group was not included in this secondary analysis of anandamide levels.

All data was collected at the Clinical Psychopharmacology Unit (University College London, London, UK). The first stage of the trial occurred from May 28, 2014, to August 12, 2015, and the second stage occurred from May 24, 2016, to January 12, 2017. The final follow-up assessment was completed on June 5, 2017, and the trial ended on May 30, 2018, due to a lack of funding for a subsequent phase 2b stage. The trial was preregistered prior to data collection with the EU Clinical Trials Register (2013-000361-36) and with clinicaltrials.gov (NCT02044809). The trial was approved by the UK Health Research Authority (13/EE/0303) and the UK Medicines and Healthcare Regulatory Agency (20363/0325/001-0001). For the protocol see https://osf.io/3cbef/.

Sample sizes for this trial were informed by a pilot study testing the effects of a 1-week course of CBD on cigarette consumption in tobacco smokers who intended to quit. The effect of CBD on cigarette cessation and cannabis cessation may be comparable as some of CBD’s potential effects are non-drug specific. For example, CBD is thought to produce anxiolytic effects [11, 13] which may counteract the withdrawal effects of both cannabis and tobacco [36]. The pilot study indicated a sample size of 12 per group would be needed to provide 80% power to detect a similar effect of CBD that was seen within the pilot study (Cohen’s *d* = 1.21). Given the uncertainty around these estimates, a sample size of 12 per group (interim analysis) and 24 per group (final analysis) was planned for the two-stage adaptive design. The interim analysis allowed for the earlier detection of inefficacious dosages (200 mg CBD/day) which were then eliminated from the trial. Subsequently, recruited participants were assigned to the remaining groups, increasing trial efficiency and allowing for a greater sample size at the final analysis. In this paper, we only report data from the final analysis.

### Participants

Participants were recruited through advertisements on websites and forums, as well as flyers in the local community. Inclusion criteria were as follows: aged 16–60 years, meeting DSM-5 criteria for a cannabis use disorder with at least moderate severity, expressing a desire to stop using cannabis and intending to do so in the next month based on an adapted Motivation to Stop scale [37], reporting at least one failed quit attempt for their cannabis use, reporting co-administering their cannabis together with tobacco, providing a urine sample positive for THC-COOH, and capacity to give informed consent as defined by Good Clinical Practice guidelines. Informed, written consent was obtained from participants before treatment began. Additionally, females of childbearing potential were required to have a negative pregnancy test within seven days of starting treatment. Females of childbearing potential and all males were also required to use an effective method of contraception from the time consent was signed until six weeks after treatment discontinuation.

Exclusion criteria were as follows: current breastfeeding or pregnancy, allergies to CBD, microcrystalline cellulose or gelatin, prescribed psychotropic drug use during screening or treatment, use of other illicit drugs more than twice a month during screening, evidence of inaccurate self-reported drug use due to a positive urine test for a drug not reported during screening, current or prior self-reported diagnosis of a psychotic disorder, any physical health problem deemed clinically significant, and not speaking English.

### Study intervention

Drugs used in the trial were contained in identical gelatin capsules containing microcrystalline cellulose filler and cannabidiol (0 mg, 50 mg, 100 mg, or 200 mg). Cannabidiol was obtained from STI Pharmaceuticals (Brentwood, UK) and manufactured by Nova Laboratories (Leicester, UK). During treatment, participants were instructed to take two capsules twice daily to achieve the daily doses of placebo, 200 mg CBD, 400 mg CBD or 800 mg CBD. Participants attended weekly site visits at baseline (day 0) and treatment days 7, 14, 21 and 28). Adherence was assessed by self-report at weekly site visits and by return of unconsumed capsules. Participants who did not show adequate adherence on any treatment week (≥30% capsules returned or ≥30% self-reported doses missed), or did not attend a site visit within two days of the scheduled appointment, or did take concomitant psychotropic medication during treatment, were not provided further CBD capsules for the duration of the trial but continued all other aspects of the protocol. Six 30-min individual sessions of motivational interviewing were provided to all participants by trained psychologists. These were provided during screening and weekly site visits. Motivational interviewing is a talking therapy that aims to help clients increase their readiness for change [38]. Meta-analyses indicate it is effective in treating substance use disorders, including cannabis use disorder [39], and this psychological intervention was provided in conjunction with CBD as clinical guidelines state that drug misuse treatment should always involve a psychosocial component [40].

### Outcome measure of plasma anandamide levels

Levels of anandamide were measured at baseline (day 0) and treatment days 14 and 28. These were determined by liquid chromatography-tandem mass spectrometry, conducted by ABS laboratories (Hertfordshire, UK). In order to extract plasma to assess plasma anandamide levels, blood was obtained using a needle and a lithium heparin anticoagulant vacuum. Plasma was obtained by immediately centrifuging blood samples using a Clifton centrifuge for five minutes at 2800 rpm, before immediate storage at −80 °C. Through this method, the lower limit of quantification for anandamide levels in plasma was 0.1 ng/ml.

### Clinical outcome measures for associations with plasma anandamide levels

Cannabis use was assessed via two different measurements which were the primary outcomes in the original clinical trial publication [14]. First, as THC:COOH-creatinine ratios (a continuous measure of cannabis use) and second, as days per week abstinent from cannabis. These two measurements were analysed separately. Measurements of urine were taken in order to assess THC:COOH-creatinine ratios, via liquid chromatography-tandem mass spectrometry conducted by ABS laboratories of urine samples taken at baseline (day 0), day 14 and day 28. Cannabis withdrawal, anxiety and depression were secondary outcomes in the original clinical trial publication [14]. Days per week abstinent from cannabis were determined using the Timeline Follow-back Method in the past seven days [41] and total scores on the Cannabis Withdrawal Scale [42], and total scores on the Beck Anxiety Inventory [43] and Beck Depression Inventory [44] were recorded during treatment weeks.

### Statistical analyses

In order to investigate the effects of CBD on anandamide levels, linear mixed-effects models were used due to the ability to incorporate missing data into analysis via maximum likelihood estimation, in an intention-to-treat analysis. Anandamide level was the dependent variable. CBD (placebo, 400 mg/day, 800 mg/day; reference category: placebo) and time (day 0, day 14 and day 28; reference category: day 0) were added as predictor variables. Because this was a clinical trial for the treatment of cannabis use disorder and changes in cannabis use could potentially influence anandamide levels, models were run both unadjusted and adjusted for cannabis use (urinary THC:COOH/creatinine ratios). CBD, time and their interaction were modelled as fixed effects. In adjusted models, cannabis use (measured as THC:COOH-creatinine ratios) was fitted as a covariate. Participant was fitted as a random intercept. The covariance structure was selected as first-order autoregressive as this provided the greatest model fit and accounted for the likelihood that measurements taken closer in time would be more strongly correlated. Significance was assessed according to an alpha level of 0.05. Models were conducted on an intention-to-treat basis under the assumption that missing data were at random. A total of 70 participants were included in the analysis. Datapoints of anandamide were missing in 21 instances. Additionally, in 11 instances, levels of anandamide were below the limit of quantification. These data were coded as missing allowing them to be estimated via maximum likelihood estimation without listwise deletion.

The original data produced a model with residuals that deviated from normality (skewness = 2.16, excess kurtosis = 6.68). Following recommendations by Tabachnick and Fidell [45], datapoints of anandamide were winsorised to minimise the influence of outlying data and facilitate normally distributed residuals. Datapoints of anandamide were stratified by CBD dosage and time, then 9 values above or below two standard deviations from the within-group mean were winsorised with values at the two standard deviation threshold. This reduced deviations from normality in the residuals to within recommended bounds (skewness = 1.29, excess kurtosis = 1.95) [46]. Results of the linear mixed-effects model are reported using winsorised data.

In order to investigate the extent to which changes in anandamide levels were associated with changes in anxiety, depression, cannabis use or withdrawal, Spearman’s rank-order correlation coefficients were calculated. This was done using the winsorised data. Correlations were stratified by CBD dosage and were conducted between changes in anandamide levels (from day 0 to 28) and changes in anxiety/depression/cannabis use (as THC:COOH-creatinine ratios or as days per week abstinent from cannabis)/cannabis withdrawal (from day 0 to 28). Significance was assessed using Bonferroni-adjusted alpha levels of 0.0125 (0.05/4). Bias corrected and accelerated bootstrapped confidence intervals of correlation coefficients were calculated based on 10,000 samples. All analyses were conducted using IBM SPSS version 27.

## Results

Participant characteristics are shown in Table 1. Drop-out rates throughout the trial were low, with two participants from the placebo group and one participant from the 400 mg CBD/day group not receiving the allocated treatment (Fig. 1).

**Table 1:** Participant characteristics within treatment groups.

	Placebo	400 mg/day CBD	800 mg/day CBD
*n*	23	24	23
*Age (in years)*			
*M*	24.9	26.6	27.4
*SD*	7.4	6.8	5.8
Range	18–45	18–47	19–38
*Sex*			
Male	17 (74%)	17 (71%)	16 (70%)
Female	6 (26%)	7 (29%)	7 (30%)

**Fig. 2:**
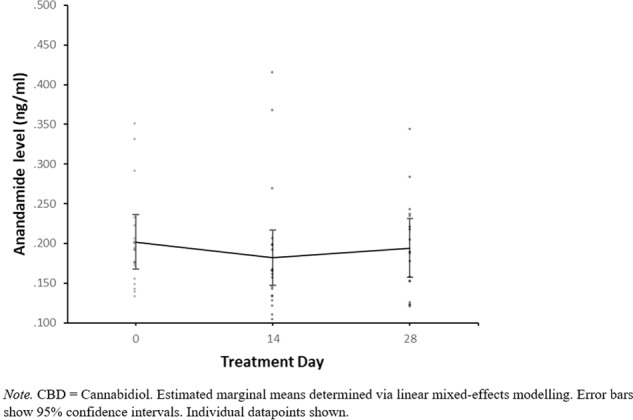
Estimated marginal means of anandamide levels in the 400 mg CBD group by week.

**Fig. 3:**
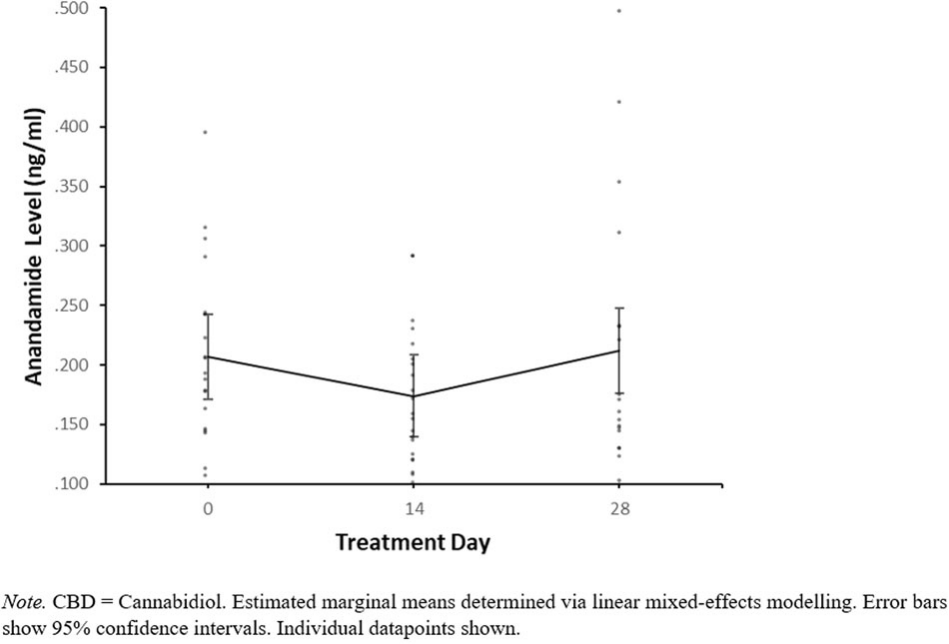
Estimated marginal means of anandamide levels in the 800 mg CBD group by week.

**Fig. 4:**
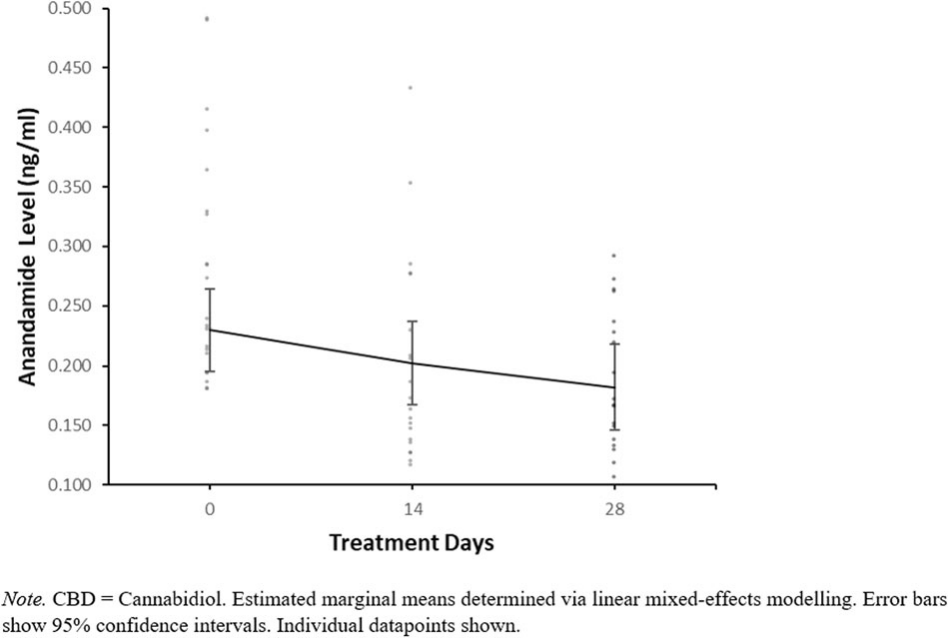
Estimated marginal means of anandamide levels in the placebo group by week.

**Fig. 5:**
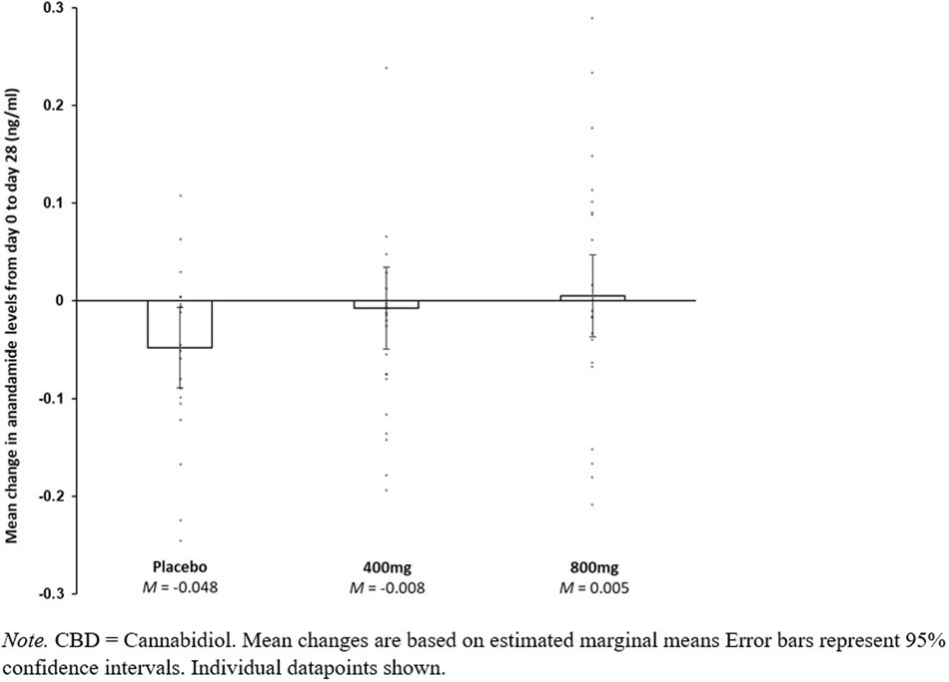
Within-group changes in anandamide levels from day 0 to day 28.

There was no evidence for a CBD*time interaction with 400 mg CBD compared to placebo in either the adjusted or unadjusted models, indicating that 400 mg CBD did not significantly affect anandamide levels relative to placebo. For 800 mg CBD vs placebo there was evidence for a CBD*time interaction from day 0 to day 28 in the adjusted model (*p* = 0.046) but not the unadjusted model. In order to investigate this CBD*time interaction further, post hoc pairwise comparisons were conducted in the adjusted model to compare anandamide levels at day 0 compared to day 28, stratified within the placebo and 800 mg CBD groups. In the placebo group, anandamide levels were higher at day 0 (Mean = 0.230, SE = 0.017) than day 28 (Mean = 0.182, SE = 0.018), *p* = 0.042 (following Bonferroni adjustment for multiple post hoc pairwise comparisons), with a mean difference of −0.048, 95% CI [−0.089, −0.007]. In contrast, in the 800 mg CBD group anandamide levels at day 0 (Mean = 0.207, SE = 0.018) were not significantly different from those at day 28 (Mean = 0.212, SE = 0.018), *p* = 1.000 (following Bonferroni adjustment), with a mean difference of 0.005, 95% CI [−0.036, 0.047]. Anandamide levels at days 0, 14 and 28 can be visualised within individual CBD dosage groups in Figs. 2, 3 and 4, while changes in anandamide levels from days 0 to 28 can be seen in Fig. 5.

### Association between changes to anandamide levels and changes to clinical outcomes

Changes in anandamide levels between day 0 and day 28 were not significantly correlated with changes to anxiety, depression, cannabis use (as THC:COOH-creatinine ratios or days per week abstinent from cannabis) or withdrawal symptoms between day 0 to day 28, within CBD and placebo groups (all *p* values > .05). Fisher’s r to z tests found no significant differences in correlation coefficients between placebo, 400 mg CBD and 800 mg CBD groups when correlating changes to anandamide levels with changes to anxiety, depression, cannabis use or withdrawal symptoms.

### Adverse effects

No severe adverse effects were recorded during the trial, and no differences in mild and moderate adverse effects were observed across treatment groups.

## Discussion

In this randomised clinical trial of CBD for the treatment of cannabis use disorder, we compared the effects of 28-day treatment with 400 mg and 800 mg CBD per day to placebo on anandamide levels in plasma. After adjusting for cannabis use during the trial, we found evidence for an effect of 800 mg CBD compared to placebo on plasma anandamide levels recorded at baseline (day 0) compared to the end of treatment (day 28). Post hoc tests revealed that anandamide levels remained stable in the 800 mg CBD group from day 0 to day 28, while they significantly decreased in the placebo group. There was no evidence for an effect of 400 mg CBD compared to placebo on plasma anandamide levels. Lastly, changes to anandamide were not significantly correlated with changes to cannabis use, withdrawal symptoms, anxiety or depression in any of the three treatment groups.

These findings build upon previous work by Leweke et al. [8], which found a 28-day course of 800 mg CBD/day to increase anandamide levels when compared to baseline, or a parallel group receiving 800 mg/day of the antipsychotic amisulpride. The current study did not find 800 mg CBD increased anandamide levels compared to baseline. However, in the adjusted model, 800 mg CBD appeared to protect against reductions to anandamide levels which were experienced over the course of treatment within the placebo group. These results may therefore corroborate Leweke et al. [8] supporting an effect of 800 mg in increasing anandamide signalling relative to placebo. However, comparisons between studies must be interpreted cautiously due to sample differences – participants in Leweke et al. [8] had a diagnosis of schizophrenia and were excluded if using cannabis, while conversely in the current study, participants met criteria for cannabis use disorder and were excluded if experiencing a psychotic disorder. Given the importance of the endocannabinoid system in psychiatric disorders, future work will be necessary to evaluate the effects of repeated CBD doses on anandamide levels in other indications, as well as in healthy controls.

The finding of a reduction in anandamide levels over the 28-day-treatment period in the placebo group was unexpected, but illustrates the value of a matched placebo group when interpreting effects of CBD in this clinical population. The start of the treatment period corresponded with the beginning of a cannabis cessation attempt in all participants, which may be a possible explanation for this reduction in anandamide. Previous evidence shows that chronic cannabis use is associated with downregulation of CB1 receptors, and that this effect is reversed (an increase in CB1 receptor density) during abstinence [27, 29]. As all participants in this trial were engaged in a cannabis cessation attempt, reductions in cannabis use may have caused changes in the endocannabinoid system such as increased CB1 receptor density, thus potentially reducing the demand for anandamide, as measured in placebo-treated participants in this trial. Further research is warranted to aid the interpretation of changes in the endocannabinoid system in relation to cannabis use disorder, such as comparing changes in anandamide levels occurring during a cessation attempt versus continued use.

The key strengths of the current study include a randomised placebo-controlled and dose-response trial design. Using this, we provide the first evidence of CBD’s effects on anandamide levels in psychiatric disorders within humans, using a placebo-controlled design. Moreover, we were able to assess the effect of multiple CBD dosages, which is important as CBD has previously been found to exert anxiolytic effects in an inverted-U shaped dose-response manner [47]. However, our findings should also be considered within the context of methodological limitations. Anandamide levels were assessed in plasma, and not in cerebrospinal fluid. Although it is likely that the effect of CBD on FAAH inhibition is seen in plasma anandamide levels [8], measurements taken within cerebrospinal fluid may be a more relevant marker of anandamide signalling. Thus, it should be acknowledged that different results may have emerged if data were collected from cerebrospinal fluid. However, sampling from cerebrospinal fluid is more invasive than sampling from plasma and may be less acceptable to participants in a clinical trial or in routine clinical practice. Additionally, anandamide is believed to be synthesised on demand in a time- and context-dependent manner [48]. Therefore, fortnightly measurements in the laboratory may have been unable to capture the effects of CBD on anandamide levels produced on demand in the context of addiction (e.g. when participants are exposed to cues that might precipitate craving and drug use). Other factors (such as contraception and/or menstrual cycle) may have influenced fluctuations in anandamide levels [49]. Given the modest sample size of this clinical trial and the preliminary nature of the findings, they should be interpreted cautiously until replicated. Furthermore, given that this trial was conducted in people meeting criteria for cannabis use disorder, this may have influenced the effects of CBD on anandamide levels and the results generated should not be generalised to other psychiatric disorders.

In summary, this study assessed the effect of CBD vs placebo on anandamide levels among individuals meeting criteria for cannabis use disorder undergoing a cannabis cessation attempt. After adjusting for cannabis use during the trial, anandamide levels significantly reduced in the placebo group across the trial, though this effect was buffered in the 800 mg CBD group, for whom anandamide levels remained stable during treatment. Taken together, these findings provide some support for the role of CBD in enhancing anandamide signalling, although further testing is needed. However, we did not find evidence that changes in anandamide levels were associated with changes in cannabis use, cannabis withdrawal, anxiety or depression during treatment. Further research in other clinical populations and sampling from cerebrospinal fluid could advance our mechanistic understanding of CBD’s effects in the treatment of psychiatric disorders.
